# Record of Fastest Time Made by Horses

**Published:** 1893-12

**Authors:** 


### Record of Fastest Time Made by Horses.

### RUNNING.*

| MILES. | | |
|---|---|---|
| ¼ | Bob Wade, 4, Butte, Mont., Aug. 20, 1890 | 0.21¼ |
| ⅜ | Fashion, 4, Lampas, Texas, Aug, 15, 1891 | 0.34 |
| ½ | Geraldine, 4, 122 lbs., N, Y. J. C., straight course, Aug. 30, 1889 | 0.46 |
| | April Fool, 4, 122 lbs., Butte, Mont., July 31, 1891 | 0.47 |
| 4½ fur. | Toano, 6, 116 lbs., Guttenburg, Jan. 8, 1892 | 0.54 |
| | Geraldine, a, 115 lbs., Oakland, Cal., Jan. 21, 1893 | |
| ⅝ | Dr. Hasbrouck, 4, 122 lbs,, N. Y. J. C., Oct. 1, 1892 | 0.57 |
| | Correction, 5, 119 lbs., N. Y. J. C., Sept. 29, 1893 | |
| 5½ fur. | Tormentor, 6, 121 lbs., N. Y. J. C., Oct. 10, 1893 | 1.03 |
| Futurity course, 170 ft. less than ¾ mile, | Kingston, a, 139 lbs., Coney Island J. C., June 22, 1891 | 1.08 |
| ¾ | Domino, 2, 128 lbs., N, Y. J. C., Sept. 29. 1893 | 1.09 |
| 6½ fur. | Wampezo, 3, 93 lbs., Guttenburg, Sept. 7, 1893 | 1.20½ |
| ⅞ | Kingston, a, 128 lbs., Monmouth Park, July 12, 1892 | 1.26 |
| | Little Billie, 4, 96 lbs . N. Y. Jockey Club, Sept. 30, 1894 | |
| | Bella B., 5, 103 lbs., Monmouth Park, July 8. 1890, straight course. | 1.23½ |
| 1 | Salvator, 4, 110 lbs., Monmouth Park, Aug. 28, 1890, against time, straight course | 1.35½ |
| | Kildeer, 4. 91 lbs., Monmouth Park, Aug. 13. 1892, straight course. | 1.37¼ |
| | Chorister, 3, 112 lbs., New York Jockey Club, June 1, 1893 | 1 39¼ |
| 1m. 20 yds. | Maid Marian, 4, 101 lbs., Chicago, Wash. Park, July 19, 1893 | 1.40 |
| 1m. 70 yds. | Wildwood, 4, 115 lbs., Chicago. Wash. Park. July 5, 1893. | 1.44 |
| | Faraday, 4. 102 lbs., Chicago, Wash. Park, July 21, 1893 | |
| 1 1-16. | Yo Tambien. 3, 99 lbs., Chicago, Wash Park, July 19, 1892 | 1.45½ |
| 1⅛ | Tristan, 6, 114 lbs , New York Jockey Club, June 2, 1891 | 1.51¼ |
| 1 3-16. | Rudolph, 5, 107 lbs., Chicago, Wash. Park, July 15, 1893 | 1.59¾ |
| | Lorenzo, 4, 104 lbs., Chicago, Garfield Park, Ang. 12, 1892 | |
| 1¼ | Salvator, 4, 122 lbs., Sheepshead Bay, June 25, 1890 | 2.05 |
| | Morello, 3, 117 lbs., Chicago, Wash. Park, July 22, 1893 | |
| | Banquet. 3, 108 lbs., Monmouth P'k, July 17, 1890, straight course | 2.03¾ |
| 1m. 500 yds. | Bend Or, 4, 115 lbs., Saratoga, July 25, 1882 | 2.10½ |
| 1 5-16. | Sir John, 4, 116 lbs., New York Jockey Club, June 9, 1892 | 2.14¼ |
| 1⅜ | Ormie, 4, 105 lbs., Chicago, Washington Park. July 7, 1890 | 2.20¼ |
| 1½ | Lamplighter, 3. 109 lbs., Monmouth Park, Aug 9, 1892 | 2.32¾ |
| 1⅝ | Hindoocraft, 3, 75 lbs., New York Jockey Club, Aug. 27, 1889 | 2.48 |
| 1¾ | Hotspur, 5, 117 lbs . San Francisco, April 30, 1891 | 3.00¾ |
| 1⅞ | Enigma, 4, 90 lbs., Sheepshead Bay, Sept. 15, 1885 | 3.20 |
| 2 | Ten Broeck, 5, 110 lbs., Louisville, May 29, 1877, against time. | 3.27½ |
| | Newton, 4, 107 lbs., Chicago, Washington Park, July 13, 1893. | |
| 2⅛ | Monitor, 4, 110 lbs , Baltimore, Oct. 20, 1880. | 3.44½ |
| 2¼ | Springbok, 5, 114 lbs., Preakness, a, 114 lbs., Saratoga, July 29, 1875 | 3.56¼ |
| 2½ | Aristides, 4, 104 lbs., Lexington, May 13, 1876 | 4.27½ |
| 2⅝ | Ten Broeck, 4, 104 lbs., Lexington, Sept. 16, 1876 | 4.58½ |
| 2¾ | Hubbard, 4, 107 lbs., Saratoga, Aug. 9, 1873 | 4.58¾ |
| 3 | Drake Carter, 4, 115 lbs, Sheepshead Bay, Sept 6, 1884 | 5.24 |
| 4 | Ten Broeck, 4, 104 lbs., Louisville, Sept. 27, 1876, against time | 7.15¾ |
| | Fellowcraft, 4, 108 lbs., Saratoga, Aug. 20, 1874 | 7.19½ |

* Goodwin's Official Turf Guide.

### HEAT RACES.

| Distance | Horse, etc. | Time |
|---|---|---|
| ¼ | Sleepy Dick, a, Kiowa, Kan., Nov., 24, 1888 | 0.21½—0.22¼ |
| ½ | Eclipse, Jr., 4, Dallas, Texas, Nov. 1, 1890 | 0.48—0.48—0.48 |
| | Bogus, a, 113 lbs., Helena, Mont., Aug. 22, 1888 | 0.48—0.48 |
| ⅝ | Kittie Pease, 4, Dallas, Texas, Nov. 2, 1887 | 1.00—1.00 |
| | Fox, 4, 113 lbs., San Francisco, Cal., Oct. 31, 1891 | 1.00 3-5—1.01 1-5 |
| ¾ | Lizzie S., 5, 118 lbs., Louisville, Sept. 28, 1883 | 1.13¼—1.13¼ |
| | Tom Hayes, 4, 107 lbs., New York Jockey Club, June 17, 1892, straight course | 1.10½—1.12¾ |
| 1 | Guido, 4, 117 lbs., Chicago, Wash. Park; July 11, 1891 | 1.41½—1.41 |
| 1 | 3 in 5. L'Argentine, 6, 115 lbs., St. Louis, June 14, 1879 | 1.43—1.44—1.47¾ |
| 1 1-16 | Slipalong, 5, 115 lbs., Chicago, Wash. Park, Sept. 2, 1885 | 1.50½—1.48 |
| 1⅛ | Gabriel, 4, 112 lbs., Sheepshead Bay, Sept. 23, 1880 | 1.56—1.56 |
| 1¼ | Glenmore, 5, 114 lbs., Sheepshead Bay, Sept. 25, 1880 | 2.10—2.14 |
| 1½ | Patsy Duffy, a, 115 lbs., Sacramento, Cal., Sept. 17, 1884 | 2.41¾—2.41 |
| 2 | Miss Woodford, 4, 107½ lbs., Sheepshead Bay, Sept. 20, 1884 | 3.33—3.31¼ |
| 3 | Norfolk, 4, 100 lbs., Sacramento, Sept. 23, 1865 | 5.27½—5.29½ |
| 4 | Ferida, 4, 105 lbs., Sheepshead Bay, Sept. 18, 1880 | 7.23½— 7.41 |

---

### TROTTING—ONE MILE.

| Age | Horse, etc. | Time |
|---|---|---|
| Yearling. | Pansy McGregor, f. [race record], Nov. 18, 1893 | 2.23¾ |
| 2-year-old. | Arion, b.c., Stockton, Cal., Nov. 10, 1891, against time | 2 10¾ |
| | Silicon, f., 1893 | 2 15¾ |
| 3-year-old. | Arion, c., 1892 | 2.10½ |
| | Fantasy, f., 1893 | 2.08¾ |
| 4-year-old. | Directum, b'k h., Lexington, Ky. [race record], 1893 | 2.05¼ |
| | Alix, m., 1892 | 2.10 |
| Aged. | Nancy Hanks, m., Terre Haute, Ind., Sept. 28, 1892, time record | 2.04 |

### TWO MILES.

Greenlander, h., Terre Haute, Ind., Nov. 4, 1893 ............ 4.32

### THREE MILES.

Nightingale, m., 1893 ............ 6 55½

### FIVE MILES.

Pascal, g., aged, Fleetwood, 1893 ............

### TEN MILES.

Pascal, g., aged, Fleetwood, 1893 ............ 26.15

---

### PACING.

| Horse | Time |
|---|---|
| Direct, h., aged, 1892 | 2.05½ |
| Mascot, g., aged, 1892 | 2.04 |
| Flying Jib, g., aged, 1893 | 2.04 |
| May Marshall, m., 1893 | 2.08¼ |

